# Successful Reintegration of a Golden‐Headed Lion Tamarin ( *Leontopithecus chrysomelas* ) Sheds Light on Management of This Endangered Species—Case Report

**DOI:** 10.1111/jmp.70063

**Published:** 2026-02-09

**Authors:** Danilo Simonini‐Teixeira, Ana Vitoria Dias do Nascimento, Luis Fernando de Mattos Moreira, Geovana Pôrto de Oliveira, Fernanda Silva Lima, Júlia Souza Viana, Mariana Soares da Silva, Joanison Vicente Dos Santos Teixeira, Dominic Wormell, Leonardo de Carvalho Oliveira

**Affiliations:** ^1^ State University of Santa Cruz – Ilhéus BA Brazil; ^2^ Graduate Program in Zoology State University of Santa Cruz Ilhéus BA Brazil; ^3^ Graduate Program in Animal Science State University of Santa Cruz Ilhéus BA Brazil; ^4^ Tamarin Trust London UK; ^5^ Graduate Program in Ecology and Biodiversity Conservation State University of Santa Cruz Ilhéus BA Brazil; ^6^ Bicho Do Mato Instituto de Pesquisa Belo Horizonte MG Brazil

**Keywords:** atlantic forest, post‐release monitoring, rehabilitation, wildlife rescue

## Abstract

A male *Leontopithecus chrysomelas* rescued after being found in a road in Bahia, Brazil, received clinical treatment and was successfully reintegrated into its native group after 49 days. Post‐release monitoring confirmed normal behavior, highlighting the importance of coordinated field–captive management for endangered primate rehabilitation.

Reintroduction is the intentional movement and release of an organism that has disappeared from its indigenous range and aims to re‐establish a viable population of a focal species that is locally extinct within its original range [1]. Two types of releases may be used in a reintroduction program: soft and hard releases. Soft release is based on the delayed release of individuals from a temporary enclosure, giving them time to adjust to their new environment, while hard release involves the immediate and direct release of individuals without any previous acclimatization [2]. Reintegration is the process of returning individuals to their native group, which may need to follow the initial release.

The golden‐headed lion tamarin ( *Leontopithecus chrysomelas* ) is an endangered [3] species endemic to the Atlantic Forest of Brazil, currently restricted to the state of Bahia [4]. Over the past 30 years, the species has lost 42% of its geographical distribution and around 59% of its population size, due largely to habitat loss and the effects of habitat fragmentation [5], which include road kills, contact with domestic animals, and illegal trafficking. In the past two years, many individuals have been found dead in peri‐urban and urban areas as a result of roadkill (4), electrocution (3), and domestic animal attacks (3), in addition to many records of individuals using trees and high‐voltage wires inside the city of Ilhéus [6].

On September 2, 2024, an adult male golden‐headed lion tamarin was found near the BR‐101 highway, adjacent to the Serra das Lontras National Park (Figure 1), coordinated by the Chico Mendes Institute of Biodiversity in the municipality of Arataca (39°10′24.14″W / 14°47′48.66″S). The animal was rescued by a passerby who placed it in a cardboard box and took it to the park headquarters. Upon arrival, he reported to the staff that he had observed other individuals from the same group as the injured animal waiting for it to continue its foray into the forest. Our field assistant kept track of the non‐habituated group (composed of eight individuals) while the injured animal was subsequently transported to the Wildlife Research and Care Centre (NAPAS), a section of the Veterinary Hospital in the city of Ilhéus, 200 km from where it was found.

**FIGURE 1:**
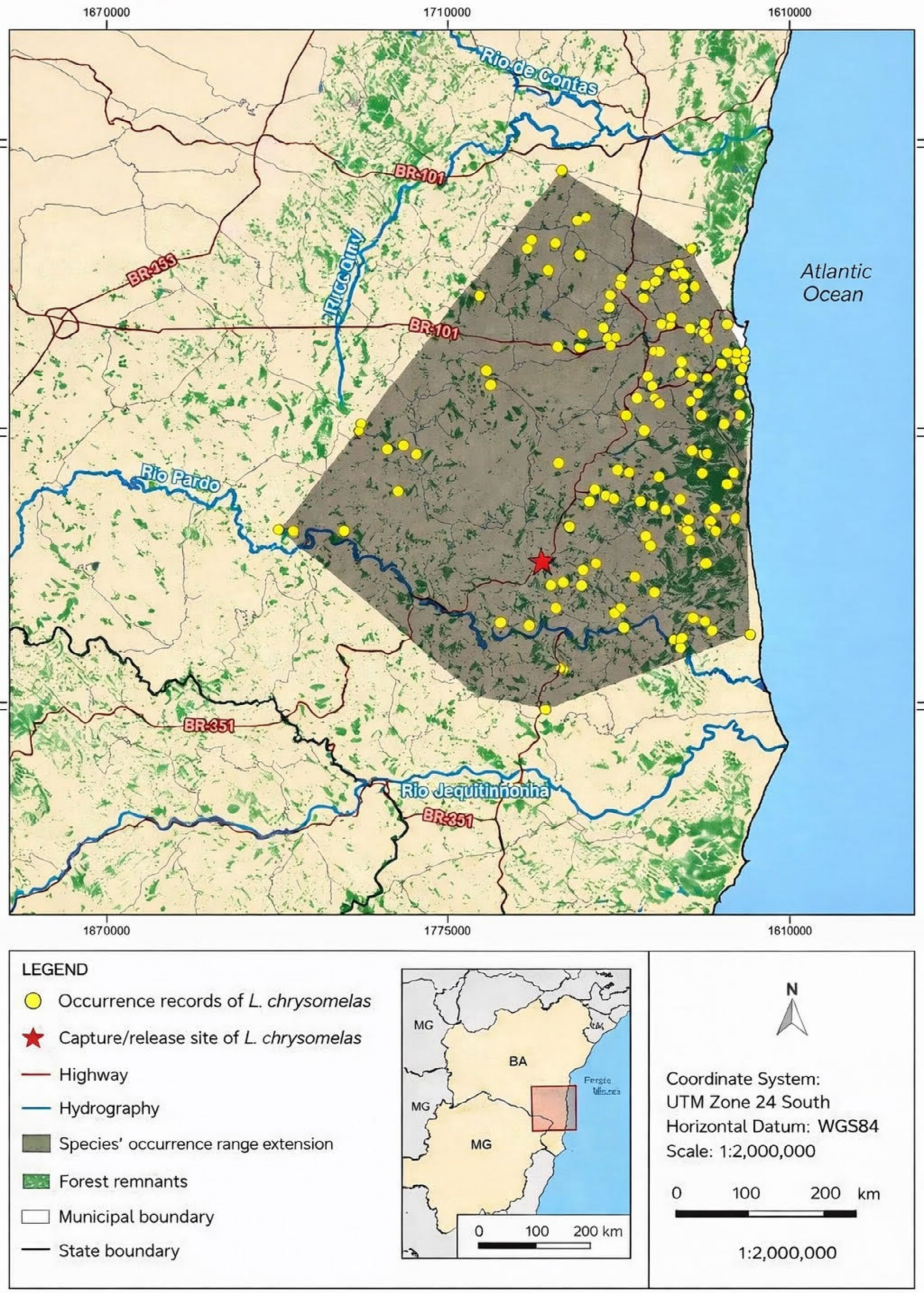
A golden‐headed lion tamarin ( *Leontopithecus chrysomelas* ) in its geographic range. Photo: Joanison V. S. Teixeira.

At NAPAS, a clinical evaluation was performed, including inspection of the head, limbs, body, and thoracic and abdominal palpation. The physical and imaging examinations did not reveal any fractures, ruling out the initial suspicion of a road accident. The animal presented physiological parameters within normal limits (heart rate: > 200 bpm, normal‐colored mucus membranes, good body condition). Hematological exams (blood count and serum biochemistry) were performed, which also showed normal results, ruling out the possibility of infectious diseases [7, 8]. Hematological examination revealed values within the reference ranges for males of the species. Erythrocyte count was 5.8 × 10^6^/μL (reported range: 5.9–6.1 × 10^6^/μL), leukocyte count was 5.6 × 10^3^/μL (reported range: 5.4–5.8 × 10^3^/μL), hematocrit was 46% (reported range: 41.2%–48%), and total plasma protein was 7.0 g/dL (reported range: 6.4–8.0 g/dL), supporting the absence of hematological alterations compatible with infectious diseases [7, 8]. However, the animal was unconscious, showed no movement, and only the head and eyes exhibited slow activity. It was hypothermic (36°C), dehydrated, and hypoglycemic, suggesting a traumatic brain injury.

On the same day, treatment was initiated with dexamethasone (1 mg/kg) and a non‐steroidal anti‐inflammatory drug for 5 days; tramadol (4 mg/kg) for analgesia, also for 5 days; and Ringer's Lactate (30 mL/kg) for hydration, in addition to mineral and vitamin supplementation (Glicopan and Bionew).

The treatment was based on the symptoms and tests performed. First, there was a need to rehydrate the animal, calculated according to its weight (600 g) and level of dehydration [9]. Concurrently, SAIDs (dexamethasone) were administered to reduce inflammation [10], and analgesics (tramadol) were given to alleviate pain [11]. Mineral and vitamin supplementation is important for maintaining the body's homeostatic balance, such as the use of B complex in cases of trauma, as they have an analgesic effect on neuropathic and nociceptive pain syndromes [12, 13]. Since the animal was initially unresponsive, it was placed in a 50 × 30 × 20 cm cage to limit movement and maintain body temperature.

Improvement in the tamarin's clinical condition was observed from the third day of treatment. The animal's temperature and hydration were reestablished, and it showed interest in its diet (150 g of fruit and one egg, twice a day). It also began to regain body movement, though with some difficulty. While it could stand and maintain posture, its head was tilted to one side, and it exhibited circular movements to the right, with a lack of sensitivity in the right limbs (the arm was falling and dragging the hand), indicative of traumatic brain injury.

After 5 days of initial treatment without improvement in neurological signs (walking in circles and numbness in the limbs), it was decided to use pentoxifylline (4 mg/kg) [14]. This drug is employed as a peripheral vasodilator, improving blood circulation and perfusion, and consequently enhancing body oxygenation. It is indicated for the treatment of vascular diseases, claudication, and even for wound healing [15, 16]. Pentoxifylline was administered for seven consecutive days. After showing improvement within the first few days following drug administration, the individual was transferred to a larger enclosure (90 × 100 × 50 cm) equipped with a nest box, branches to encourage movement and spatial exploration, and bowls for food and water. The larger enclosure facilitated behavioral evaluation of progress (movement, jumping, climbing, foraging, etc.). By the third day of use, the symptoms had resolved, demonstrating the effectiveness of the treatment.

Although we cannot rely on more sophisticated diagnostic tests, such as tomography or magnetic resonance imaging, complementary tests—including clinical history, physical examination, hematological tests (blood count and serum biochemistry), radiographs, and microbiological tests—ruled out any other type of involvement, aside from traumatic brain injury [17]. The clinical signs observed during the initial examination, such as myoclonia, lack of sensitivity and movement in the limbs, hypothermia, dehydration, bradycardia, and bradypnea, corroborate the findings of Sande et al. (2016) [18]. Additionally, the circular displacement to the right and the lack of sensitivity in the right limbs (arm and leg) suggest brain trauma [19]. Since no fractures were diagnosed during the physical examination or through radiographic imaging (Fujifilm, FCR CR—IR391RU, PRIMA 2), the initial hypothesis of a run‐over was ruled out. However, upon analyzing the area where the animal was later found, we propose that the traumatic brain injury was caused by a jump from one tree to another with the intention of crossing the road.

By September 15, 2024, the animal weighed 700 g and exhibited completely restored behavior. Physiological parameters, including heart rate, respiratory rate, and body temperature, returned to reference values [7, 8]. The animal resumed normal feeding behavior and exhibited adequate motor activity within the enclosure, including jumping, climbing, and moving between structures.

On September 26, 2024, a microchip (number: 9633001181853) was implanted for identification. On October 21, 2024, a radio collar (Holohil RI‐2D) was fitted, and the animal was released at the site where it was found (a hard release). Monitoring in the field showed that the group seen close to where the individual was first observed had continued to use the same area and had the same composition as before. As lion tamarins are territorial, this suggested that the individual had come from this group [3]. We tracked the male until it rejoined its native group in two days. We continued to monitor the group throughout complete days (from when they left their sleeping site in the morning until they returned in the evening) or partial days (either from the time they left the sleeping site until noon or from noon until they entered a sleeping site), following the methods used in ecological studies for the species [4, 5]. We monitored the entire group for two months (daily on the first week after release and then once a week) and recorded affiliative social interactions, such as grooming, resting while in contact with one another, food sharing, and playing. These behaviors are commonly observed in tamarins [20] and are frequently used as a measure of affiliation by members of a group [21]. We observed the reintegrated animal participating in all affiliative social interactions (Figure 1) [20, 21] and we considered he was accepted back to its native group, suggesting that the reintegration was successful [22].

This tamarin was under human care for almost 50 days since the day it was found lying on the ground. Rapid and thorough care in captivity resulted in fast recovery, and consequently a hard release was possible. During his period in captivity, the tamarin was fed fruits that did not match its natural diet and underwent procedures that required direct and constant contact with humans, but was able to go back into the wild without problems. Furthermore, even though the individual was outside its group and still in direct contact with humans, it was possible to reestablish its contact with its original group with relative ease. Monitoring the group during the individual's absence meant that we were able to release him into their home range and make it more likely that he would rejoin the group.

However, it is important to note that the success of this program of treatment and reintroduction may not be reproducible in every case. The position of an animal in its group, the composition of the group as a whole, the nature of its injuries, and the time an animal is away from its group are only some of the factors that may influence the success of such initiatives. Close monitoring of both of the rescued individual and its group, as was done in this case, is essential.

## Ethics Statement

The authors confirm that this study was performed in accordance with the ethical policies of the journal, as noted on the journal's author guideline page.

## Conflicts of Interest

The authors declare no conflicts of interest.

## Data Availability

Data sharing not applicable to this article as no datasets were generated or analysed during the current study.
